# Body Mass Index and Survival in Children Receiving Extracorporeal Membrane Oxygenation

**DOI:** 10.1001/jamanetworkopen.2026.6162

**Published:** 2026-04-20

**Authors:** Pilar Anton-Martin, Keith Baxelbaum, Shilpa Vellore, Erika O’Neil, Vinai Modem, Sameer Thadani

**Affiliations:** 1 Department of Pediatrics, Division of Anesthesiology and Critical Care Medicine, Children’s Hospital of Philadelphia, Philadelphia, Pennsylvania; 2 Department of Biomedical and Health Informatics, Data Science and Biostatistics Division, Children’s Hospital of Philadelphia, Philadelphia, Pennsylvania; 3 Department of Pediatrics, Division of Cardiology, University of California, San Diego School of Medicine/Rady Children’s Hospital, San Diego; 4 Department of Pediatrics, Air Force, Department of Pediatrics, Brooke Army Medical Center, San Antonio, Texas; 5 Department of Pediatrics, Uniformed Services University of Health Sciences, Bethesda, Maryland; 6 Division of Pediatric Critical Care Medicine, Cooks Children’s Hospital, Fort Worth, Texas; 7 Division of Pediatric Critical Care Medicine and Pediatric Nephrology, Baylor College of Medicine, Texas Children’s Hospital, Houston

## Abstract

**Importance:**

Underweight and obesity represent opposite ends of the nutritional risk spectrum that negatively affect children’s health worldwide. Both conditions have been associated with increased mortality in pediatric patients receiving extracorporeal membrane oxygenation (ECMO) support.

**Objective:**

To investigate the association of underweight and obesity status with pediatric ECMO outcomes using body mass index (BMI) *z* scores.

**Design, Setting, and Participants:**

This retrospective cohort analysis used data from the Extracorporeal Life Support Organization (ELSO) database from January 1, 2019, to December 31, 2023. Participants included all pediatric patients from international ECMO centers aged 29 days to 18 years who received ECMO support during the study period. Data were analyzed from July 1, 2024 to June 30, 2025.

**Exposure:**

BMI or weight for length.

**Main Outcomes and Measures:**

Survival to hospital discharge.

**Results:**

A total of 8885 children undergoing their first ECMO run during the study period were included. The median age was 1.9 years (IQR, 0.4-10.7 years), the median weight was 11 kg (IQR, 6-36 kg), and 4773 patients (53.7%) were male. Among the cohort, 6443 patients (72.5%) had a normal weight (*z* scores −2 to 2), 1458 (16.4%) had underweight (*z* score <–2), and 984 (11.1%) had obesity (*z* score >2). Overall hospital survival occurred in 5453 patients (61.2%) but was significantly lower in patients with underweight (849 [58.2%]) and obesity (565 [57.4%]) compared with normal weight (4020 [62.4%]; *P* < .001). In multivariable logistic regression, having underweight (odds ratio [OR], 1.34; 95% CI, 1.09-1.66; *P* = .005) and obesity (OR, 1.25; 95% CI, 1.00-1.56; *P* = .04) were associated with increased hospital mortality. This pattern persisted across subgroup analyses based on age and diagnosis leading to ECMO cannulation. Severe obesity (BMI *z* score >3) was not associated with mortality.

**Conclusions and Relevance:**

In this cohort study of children receiving ECMO, 1 in 4 had an abnormal BMI, which was associated with increased hospital mortality. Prospective studies are warranted to further elucidate and define the association of nutritional status and clinical outcomes in this population.

## Key Points

**Question:**

Is body mass index (BMI) associated with outcomes in children supported with extracorporeal membrane oxygenation (ECMO)?

**Findings:**

In this cohort study of 8885 children receiving ECMO support, one-fourth were classified as having underweight or obesity. Both underweight and obesity were independently associated with higher hospital mortality.

**Meaning:**

These findings suggest that BMI-based nutritional status may help guide ECMO candidacy and nutritional management during ECMO support.

## Teaser

This cohort study investigates the association between underweight and obesity status and outcomes among children who receive extracorporeal membrane oxygenation support.

## Introduction

Malnutrition, as defined by the World Health Organization, refers to imbalances in an individual’s intake of energy and nutrients and includes both undernutrition (eg, underweight) and overnutrition (eg, overweight and obesity).^1^ Childhood obesity and malnutrition represent 2 ends of the nutritional risk spectrum that impact children globally.^1^ The obesity pandemic has accelerated during the past decades, with rising rates even among infants and young children, while undernutrition continues to persist.^1,2^ Both extremes of nutritional status are associated with increased morbidity, use of health care services, and long-term health consequences.^3,4^ In critically ill children, these vulnerabilities may be further magnified.

Several studies have demonstrated that critically ill children on either end of the body weight spectrum experience increased rates of morbidity and mortality.^4,5,6,7,8,9^ The physiological reserves of children with underweight may be inadequate to withstand the demands of critical illness, while excess adiposity in children with overweight may lead to complications associated with ventilation, medication dosing, and chronic inflammation.^4,5,6,7,8,9^ Given these associations, nutritional status is an important clinical factor used in the field of pediatric critical care.

The use of extracorporeal membrane oxygenation (ECMO) in critically ill children has increased during the past decade, establishing it as a life-saving therapy to support a variety of diseases.^10^ Despite the growing literature associating abnormal weight with poor outcomes, few studies have systematically evaluated the association between weight status and outcomes in children requiring ECMO.^11,12,13^ Building on previous work,^11,12,13^ we aimed to address this unresolved knowledge gap by leveraging the Extracorporeal Life Support Organization (ELSO) Registry to investigate the association between underweight and obesity status and pediatric ECMO outcomes.

## Methods

This retrospective cohort study used data from the ELSO Registry. Based on the Common Rule and the use of deidentified registry data, the study was exempt from review and the need for informed consent. The study followed the Strengthening the Reporting of Observational Studies in Epidemiology (STROBE) reporting guideline.

### Study Population and Definitions

All children and adolescents (hereinafter referred to as children) aged 29 days to 18 years who underwent ECMO support between January 1, 2019, and December 31, 2023, were included in the study. In alignment with ELSO recommendations, data on neonates were not requested due to the substantial weight fluctuations that commonly occur during the neonatal period. Patients were excluded from the analysis for several reasons, as detailed in eFigure 1 in Supplement 1. For patients with multiple ECMO runs, only the first run was included. We excluded patients discharged while receiving ECMO to another facility, those missing weight data, and those older than 2 years without height data. Additional exclusions included chromosomal abnormalities, prematurity, malabsorption, short gut syndrome, failure to thrive, or other disorders affecting nutrition, metabolism, fluid balance, or electrolyte levels. To minimize the impact of possible data entry errors and/or nonphysiological values, we also excluded patients with a body mass index (BMI), weight, or height *z* score greater than 10 or less than −10.

Data included demographic information, diagnoses leading to ECMO cannulation, pre-extracorporeal life support characteristics, ECMO support details, ECMO complications, and outcomes. Race and ethnicity were not included in the dataset due to limitations within the ELSO registry in accurately capturing social determinants of health. Primary diagnoses leading to ECMO cannulation were categorized into the following groups: acute respiratory distress syndrome (ARDS); non-ARDS infectious and noninfectious respiratory failure; cardiogenic shock; heart failure, cardiomyopathy, myocarditis, or arrhythmia; postoperative cardiac; arrest or extracorporeal cardiopulmonary resuscitation; sepsis or infection; hematology or oncology; and other or unknown. Data on nutritional support practices, including the mode, timing, and composition of nutritional intake, were not available in the database.

Severity of illness indicators before ECMO initiation included pH level, hypotension, use of cardiopulmonary bypass (CPB), use of kidney replacement therapy (KRT), high-frequency oscillatory ventilation and prone positioning, pulmonary vasodilators, and vasoactive agents. Hypotension was defined as systolic blood pressure less than 2 SD below age-appropriate norms. Pulmonary vasodilators included nitric oxide, sildenafil, and epoprostenol. Vasoactive agents were grouped into inotropes (dopamine, dobutamine, epinephrine, milrinone, enoximone, and levosimendan) and vasopressors (norepinephrine, phenylephrine, and vasopressin). Complications were defined per ELSO registry data definitions.^14^

### Study Objectives

The primary objective was to explore the association between BMI grouped as underweight, normal weight, or obesity and hospital survival in children receiving ECMO. The secondary objectives were (1) to identify a specific BMI inflection point that could reliably estimate hospital survival; (2) to examine the association between both underweight and obesity and the incidence of ECMO-related complications; and (3) to investigate whether severe obesity (*z* score >3) influenced mortality.

### Primary Exposure: Nutritional Status

Weight was defined as the patient’s weight in kilograms at the time of admission to the ECMO center. Length (for patients aged ≤2 years) and height (for those aged >2 years) at admission were recorded in meters. Nutritional status was defined using weight-for-length for children 2 years or younger and BMI for children older than 2 years.^3,15^ BMI was computed as weight in kilograms divided by height in meters squared. In patients 2 years or younger with unavailable length data, weight-for-age was used as a proxy.^3,16^ To compare BMI, weight-for-length, and weight-for-age across the large heterogeneous pediatric population supported by ECMO, these variables were converted into *z* scores using the 2006 World Health Organization growth standards (aged 0-2 years) and the 2000 Centers for Disease Control and Prevention growth charts (older than 2 to 20 years).^3,17^ Patients were then categorized as having underweight (*z* score, <–2), normal weight (*z* scores, −2 to 2), or obesity (*z* score, >2).^8,15,16^ It is important to note that the term *BMI* is used throughout our report to simplify interpretation; however, in children 2 years or younger, it refers to weight-for-length (or weight-for-age when length was unavailable).

### Outcomes

The primary outcome was survival to hospital discharge. Patients who were discharged alive to home or another facility but not discharged supported by ECMO were considered survivors to hospital discharge. Secondary outcomes were length of hospital stay, duration of mechanical ventilation, and presence of ECMO-related complications.

### Statistical Analysis

Data were analyzed from July 1, 2024, to June 30, 2025. Continuous variables are reported as medians and IQRs, while categorical variables are reported as frequencies and percentages. Differences in the distribution of categorical variables were tested using Pearson χ^2^ or Fisher exact tests, and continuous variables were compared using the Wilcoxon or Kruskal-Wallis rank-sum test. The final multivariable logistic regression models included our primary exposure, clinical factors previously identified to be associated with mortality in our study population, and variables with a significance level of *P* < .05 in univariate analysis, using only complete cases. These models were adjusted for age at ECMO cannulation (coded continuously in days), diagnostic groups, severity of illness indicators at the time of ECMO initiation, specific characteristics related to the ECMO run, and other known factors associated with poorer outcomes. ECMO-related complications were coded as a binary indicator reflecting the presence of any complication noted in the ELSO database for that ECMO run. The same statistical approach was applied in a secondary analysis, which further stratified patients as those with obesity (*z* score, 2-3) and those with severe obesity (*z* score, >3). Statistical significance was set at 2-sided *P* ≤ .05.

To assess the importance of our BMI *z* score threshold (part of our data preprocessing), a sensitivity analysis across *z* score cutoffs ranging from 4 to 10 or −4 to −10 was conducted. For each threshold, participants with *z* scores more extreme than the specified cutoff for any of height, weight, or BMI were excluded. The same multivariable logistic regression model was then applied to each resulting cohort. The overall results were stable, with key variables retaining statistical significance (eTable 1 in Supplement 1). To investigate whether alternative BMI thresholds (beyond the conventional *z* scores of −2 to 2) could serve as inflection points for hospital survival, we conducted receiver operating characteristic (ROC) curve analyses within 2 groups: (1) patients with a BMI *z* score greater than 0 and (2) patients with a BMI *z* score less than 0. Inflection points were identified by optimizing the Youden index. This was conducted for unadjusted BMI and for models with full covariates. Additionally, 2 subgroup analyses (age and primary diagnosis leading to ECMO cannulation) were performed to explore the potential interaction by each of these groups on the association between BMI and mortality. All analyses were performed using R, version 4.4.0 (R Project for Statistical Computing).

## Results

### Patient Characteristics

During the study period, 11 351 patients underwent a total of 12 215 ECMO runs. After using the exclusion criteria noted above, 8885 patients undergoing their first ECMO run were available for analysis (eFigure 1 in Supplement 1). Among these, 6443 patients (72.5%) were categorized with a normal weight, 1458 (16.4%) with underweight, and 984 (11.1%) with obesity. The median age was 1.9 years (IQR, 0.4-10.7 years), and the median weight was 11 kg (IQR, 6-36 kg). Overall, 4112 participants (46.3%) were female, 4773 (53.7%) were male, and 6975 (78.5%) received VA or VVA ECMO support. Overall survival to hospital discharge occurred in 5434 patients (61.2%). Complete data for anthropometric characteristics (age, weight, sex, diagnostic group), pre-ECMO factors (hypotension, inotropic support, CPB use), ECMO characteristics (type, modality, KRT utilization), complication rates (mechanical, metabolic, hemorrhagic, pulmonary, kidney, limb-related), survival, duration of mechanical ventilation, and hospital length of stay for the 3 BMI-based groups are provided in Table 1 and eTable 2 in Supplement 1.

**Table 1.** Patient Characteristics by BMI *z* Score Category^a^

Characteristic	Patient group				*P* value
	Overall (N = 8885)	Underweight (n = 1458)	Normal weight (n = 6443)	Obesity (n = 984)	
Age, median (IQR), y	1.9 (0.4-10.7)	0.6 (0.3-2.9)	2.4 (0.5-11.5)	4.4 (0.6-13.9)	<.001
Age group					
Infant (29 d to <1 y)	3590 (40.4)	917 (62.9)	2356 (36.6)	317 (32.2)	<.001
Preschool (1 to 5 y)	2175 (24.5)	325 (22.3)	1644 (25.5)	206 (20.9)	
School-age (6 to 11 y)	1171 (13.2)	112 (7.7)	908 (14.1)	151 (15.3)	
Adolescent (12 to 18 y)	1949 (21.9)	104 (7.1)	1535 (23.8)	310 (31.5)	
Weight, median (IQR), kg	11 (6-36)	6 (4-12)	12 (7-39)	24 (8-95)	<.001
Sex					
Female	4112 (46.3)	635 (43.6)	3057 (47.4)	420 (42.7)	.001
Male	4773 (53.7)	823 (56.4)	3386 (52.6)	564 (57.3)	
CDH	26 (0.3)	10 (0.7)	13 (0.2)	3 (0.3)	.01
Diagnostic group					
ARDS	523 (5.9)	62 (4.3)	384 (6.0)	77 (7.8)	<.001
Non-ARDS respiratory failure	2051 (23.1)	290 (19.9)	1486 (23.1)	275 (27.9)	
Cardiogenic shock	541 (6.1)	98 (6.7)	386 (6.0)	57 (5.8)	
HF, CMP, myocarditis, or arrhythmia	1180 (13.3)	165 (11.3)	923 (14.3)	92 (9.3)	
Postoperative cardiac	2071 (23.3)	490 (33.6)	1433 (22.2)	148 (15.0)	
Arrest or ECPR	808 (9.1)	110 (7.5)	605 (9.4)	93 (9.5)	
Sepsis or infection	914 (10.3	103 (7.1%)	646 (10.0)	165 (16.8)	
Hematology or oncology	83 (0.9)	7 (0.5)	68 (1.1)	8 (0.8)	
Other or unknown	714 (8.0)	133 (9.1)	512 (7.9)	69 (7.0)	
Pre-ECMO data					
Ventilation modality					
Conventional	5669 (63.8)	972 (66.7)	4086 (63.4)	611 (62.1)	.09
HFOV	632 (7.1)	95 (6.5)	455 (7.1)	82 (8.3)	
Other	2584 (29.1)	391 (26.8)	1902 (29.5)	291 (29.6)	
pH level, median (IQR)	7.25 (7.11-7.35)	7.25 (7.10-7.34)	7.26 (7.12-7.35)	7.25 (7.11-7.35)	.50
No. missing	1783	295	1283	205	
Hypotension	3213 (44.4)	613 (51.5)	2312 (44.1)	288 (36.1)	<.001
No. missing	1649	268	1195	186	
KRT use before ECMO	319 (3.6)	43 (2.9)	236 (3.7)	40 (4.1)	.30
Pulmonary vasodilators	2229 (25.1)	377 (25.9)	1585 (24.6)	267 (27.1)	.20
Inotropes	5640 (63.5)	1010 (69.3)	4061 (63.0)	569 (57.8)	<.001
Vasopressors	3254 (36.6)	544 (37.3)	2377 (36.9)	333 (33.8)	.20
HFOV or prone	815 (9.2)	130 (8.9)	591 (9.2)	94 (9.6)	.90
CPB	1600 (18.0)	345 (23.7)	1148 (17.8)	107 (10.9)	<.001
ECMO data					
ECMO type					
Cardiac	4192 (47.2)	768 (52.7)	3057 (47.4)	367 (37.3)	<.001
Pulmonary	2803 (31.5)	365 (25.0)	2027 (31.5)	411 (41.8)	
ECPR	1890 (21.3)	325 (22.3)	1359 (21.1)	206 (20.9)	
ECMO mode					
VA or VVA	6975 (78.5)	1240 (85.0)	5049 (78.4)	686 (69.7)	<.001
VV or other	1910 (21.5)	218 (15.0)	1394 (21.6)	298 (30.3)	
KRT use during ECMO	2079 (23.4)	295 (20.2)	1519 (23.6)	265 (26.9)	<.001
ECMO-related complications					
Neurologic	1610 (18.1)	268 (18.4)	1180 (18.3)	162 (16.5)	.40
Mechanical	2364 (26.6)	312 (21.4)	1771 (27.5)	281 (28.6)	<.001
Metabolic	1092 (12.3)	151 (10.4)	813 (12.6)	128 (13.0)	.04
Hemorrhagic	1731 (19.5)	268 (18.4)	1303 (20.2)	160 (16.3)	.007
Pulmonary	560 (6.3)	70 (4.8)	420 (6.5)	70 (7.1)	.03
Kidney	2317 (26.1)	320 (21.9)	1705 (26.5)	292 (29.7)	<.001
Cardiovascular	938 (10.6)	134 (9.2)	689 (10.7)	115 (11.7)	.11
Infectious	71 (0.8)	14 (1.0)	52 (0.8)	5 (0.5)	.50
Limb-related	199 (2.2)	17 (1.1)	159 (2.5)	23 (2.3)	.01
Outcomes					
Survival to discharge	5434 (61.2)	849 (58.2)	4020 (62.4)	565 (57.4)	<.001
ECMO duration, median (IQR), h	119 (65-219)	118 (64-213)	119 (66-219)	125 (62-255)	.60
MV, median (IQR), d	13 (7-22)	14 (8-25)	12 (7-21)	13 (7-24)	<.001
No. missing	3804	643	2712	449	NA
LOS, median (IQR), d	34 (15-69)	41 (19-75)	33 (15-68)	32 (13-68)	<.001

Abbreviations: ARDS, acute respiratory distress syndrome; CDH, congenital diaphragmatic hernia; CMP, cardiomyopathy; CPB, cardiopulmonary bypass; ECMO, extracorporeal membrane oxygenation; ECPR, extracorporeal cardiopulmonary resuscitation; HF, heart failure; HFOV, high-frequency oscillatory ventilation; KRT, kidney replacement therapy; LOS, length of stay; MV, mechanical ventilation; NA, not applicable; VA, venoarterial; VVA, venovenoarterial; VV, venovenous.

^a^ Data are reported as No. (%) of patients unless noted otherwise.

### Survival to Hospital Discharge

In the univariate analysis, hospital survival was significantly lower in patients with underweight (849 [58.2%]) and obesity (565 [57.4%]) compared with normal weight (4020 [62.4%]; *P* < .001). Multivariable logistic regression revealed that both underweight (odd ratio [OR], 1.34; 95% CI, 1.09-1.66; *P* = .005) and obesity (OR, 1.25; 95% CI, 1.00-1.56; *P* = .04) BMI classifications were associated with increased hospital mortality. Additional factors associated with increased hospital mortality included pH level (acidosis) (OR, 0.35; 95% CI, 0.22-0.55; *P* < .001), hypotension (OR, 1.39; 95% CI, 1.18-1.63; *P* < .001), use of KRT prior to cannulation (OR, 1.68; 95% CI, 1.21-2.33; *P* = .002), KRT use during ECMO (OR, 1.50; 95% CI, 1.26-1.78; *P* < .001), VA or VVA support (OR, 1.36; 95% CI, 1.10-1.69; *P* = .004), ECMO duration (OR, 1.00; 95% CI, 1.00-1.01; *P* < .001), and the occurrence of any ECMO-related complication (OR, 2.19; 95% CI, 1.83-2.63; *P* < .001) (Table 2).

**Table 2.** Multivariable Logistic Regression Model to Explore the Association With Mortality to Hospital Discharge

Characteristic	OR (95% CI) [SE]	*P* value
Underweight	1.34 (1.09-1.66) [0.11]	.005
Obesity	1.25 (1.00-1.56) [0.11]	.04
Age	0.99 (0.98-1.00) [0.01]	.14
ARDS	0.76 (0.50-1.16) [0.21]	.20
Non-ARDS respiratory failure	0.76 (0.55-1.10) [0.18]	.15
Cardiogenic shock	0.81 (0.52-1.24) [0.22]	.33
HF, CMP, myocarditis, or arrhythmia	0.77 (0.53-1.13) [0.19]	.18
Postoperative cardiac	1.06 (0.74-1.54) [0.19]	.75
Arrest or ECPR	1.52 (0.98-2.37) [0.22]	.06
Sepsis or infection	1.01 (0.70-1.45) [0.18]	.97
Hematology or oncology	2.06 (0.97-4.51) [0.39]	.06
pH level	0.35 (0.22-0.55) [0.23]	<.001
Hypotension	1.39 (1.18-1.63) [0.08]	<.001
Congenital diaphragmatic hernia	3.64 (1.01-17.09) [0.69]	.06
Cardiopulmonary bypass	0.80 (0.62-1.02) [0.13]	.08
HFOV or prone before ECMO	0.97 (0.77-1.21) [0.12]	.76
Inotropes before ECMO	1.19 (0.99-1.43) [0.09]	.05
Vasopressor use before ECMO	1.09 (0.93-1.28) [0.08]	.29
Pulmonary vasodilator use before ECMO	1.05 (0.89-1.24) [0.08]	.54
KRT use before ECMO	1.68 (1.21-2.33) [0.17]	.002
VA or VVA support	1.36 (1.10-1.69) [0.11]	.004
KRT use during ECMO	1.50 (1.26-1.78) [0.09]	<.001
ECMO duration	1.00 (1.00-1.01) [0.001]	<.001
Presence of any ECMO complication	2.19 (1.83-2.63) [0.09]	<.001

Abbreviations: ARDS, acute respiratory distress syndrome; CMP, cardiomyopathy; ECMO, extracorporeal membrane oxygenation; ECPR, extracorporeal cardiopulmonary resuscitation; HF, heart failure; HFOV, high-frequency oscillatory ventilation; KRT, kidney replacement therapy; OR, odds ratio; VA, venoarterial; VVA, venovenoarterial.

### BMI Cutoff and Hospital Survival

**Figure 1.**
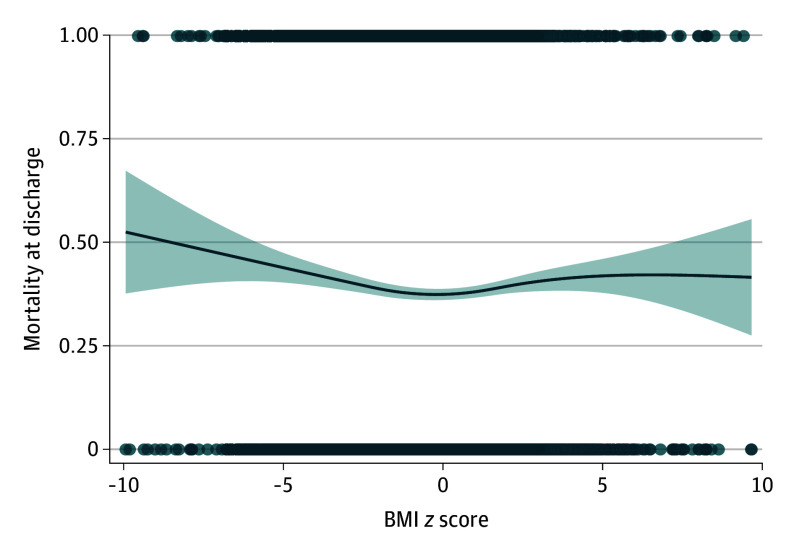
Line Graph Showing Unadjusted Association Between Body Mass Index (BMI) *z* Score and In-Hospital Mortality At the extremes, the number of patients with underweight and obesity decreased and are described as dots rather than a line.

Within the subgroup of patients with a baseline BMI *z* score less than 0, unadjusted ROC curve analysis identified an optimal cutoff at a BMI *z* score of −2.13, with an area under the ROC curve (AUROC) of 0.52. Conversely, within the subgroup with a baseline BMI *z* score greater than 0, the threshold was 1.13 (AUROC, 0.52). These AUROC values were low, indicating limited utility for defining clinically meaningful thresholds. Adjusted AUROC analyses demonstrated improved discrimination (AUROC, 0.70 for *z* scores ≥0 and −0.72 for *z* scores <0). Figure 1 visualizes a generalized additive model–based smoothed curve of unadjusted mortality probability by BMI *z* score. This exploratory visualization supports the finding of lowest association with mortality in patients with BMI in the normal range.

### Association of Severe Obesity With Mortality

When stratifying patients with obesity as those with obesity (BMI *z* score 2-3) and those with severe obesity (BMI *z* score >3), we identified significant differences across the BMI-based categories (eTables 3 and 4 in Supplement 1). In the multivariable logistic regression model, underweight status remained associated with increased hospital mortality (OR, 1.34; 95% CI, 1.09-1.65; *P* = .005), whereas neither obesity (OR, 1.26; 95% CI, 0.95-1.66; *P* = .10) nor severe obesity (OR, 1.24; 95% CI, 0.88-1.71; *P* = .21) were associated with mortality (eTable 5 in Supplement 1). These groups had smaller sample sizes (patients with obesity, n = 573; patients with severe obesity, n = 411) and similar mortality rates (244 patients with obesity [42.6%] and 175 patients with severe obesity [42.6%] in both groups compared with 2423 patients with normal weight [37.6%]), suggesting a potential limited statistical power in these analyses (eTables 3 and 4 in Supplement 1).

### Subgroup Analysis Based on Age and Diagnostic Groups

**Figure 2.**
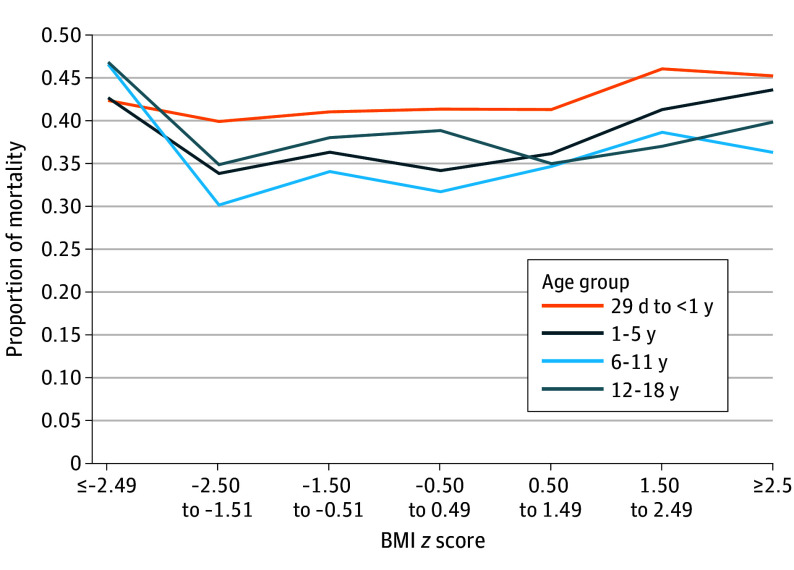
Line Graph Showing Unadjusted Proportion of Mortality by Body Mass Index (BMI) *z* Score, Stratified by Age Group

**Figure 3.**
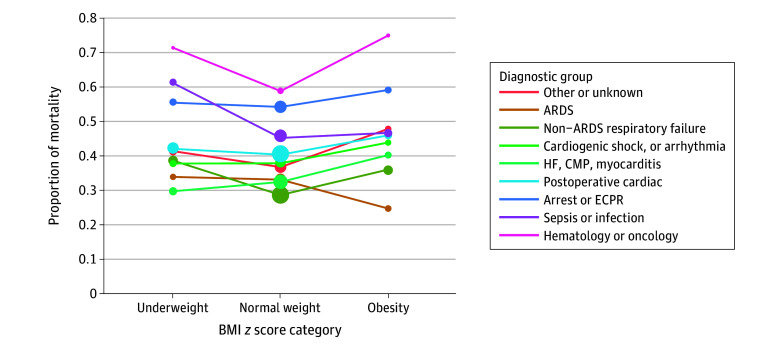
Line Graph Showing Unadjusted Proportion of Mortality by Body Mass Index (BMI) *z* Score, Stratified by Diagnostic Group Circle sizes represent the No. of patients (range, 1 [smaller circles] to 1000 [larger circles]). ARDS indicates acute respiratory distress syndrome; CMP, cardiomyopathy; ECPR, extracorporeal cardiopulmonary resuscitation; and HF, heart failure.

Age was associated with mortality with unadjusted covariates (Figure 2 and eTable 6 in Supplement 1). While the pattern of lower mortality for normal-range BMI persisted across age groups, the highest mortality proportions were noted among school-aged children with underweight (48 of 112 [42.9%]), as well as among infants (146 of 317 [46.1%]) and preschool-aged children (96 of 206 [46.6%]) with obesity. Diagnosis was also associated with mortality (Figure 3 and eTable 7 and eFigure 2 in Supplement 1), demonstrating lower mortality for the groups with normal BMI, except patients with ARDS and obesity (19 of 77 [24.7%]) and patients with underweight and heart failure, cardiomyopathy, myocarditis, or arrhythmia (49 of 165 [29.7%]).

## Discussion

In this retrospective cohort study, we evaluated the association between BMI and hospital survival in children receiving ECMO using international, multicenter data from the ELSO Registry and identified several novel insights that contribute to the existing literature. First, underweight (16.4%) and obesity (11.1%) were prevalent among children requiring ECMO, aligning with prior pediatric studies reporting rates of 15% to 33% and 5% to 9%, respectively.^11,12,13^ Second, after adjusting for age, diagnostic groups, illness severity, and ECMO-related factors known to influence outcomes, both underweight and obesity were associated with increased hospital mortality. Third, patients with obesity experienced a high proportion of ECMO-related complications, whereas patients with underweight did not. Last, severe obesity was not associated with mortality, although our sample size was limited.

In our cohort, both underweight and obesity nutritional status were associated with hospital mortality. Unadjusted AUROC analyses identified inflection points at BMI *z* scores of −2.13 and 1.13, respectively; however, the associated AUROC values were low, indicating limited discriminatory performance. Notably, a BMI *z* score between 1 and 2 refers to patients with overweight.^15^ This bidirectional pattern between both underweight and obesity status and hospital mortality is known in critically ill children but, to our knowledge, has not been reported in those receiving ECMO.^8^ While prior ECMO studies have shown increased mortality in neonates weighing less than 2 kg regardless of gestational age,^18,19,20^ data on the broader pediatric population remain limited. One pediatric single-center ECMO study^11^ found underweight status, but not obesity, to be an independent factor for hospital mortality, whereas others^12,21^ have reported no association between nutritional status and mortality.

Multiple adult ECMO studies have explored the impact of underweight and obesity on outcomes, offering physiological context for our findings. Studies focused on adults with underweight have reported no difference,^22^ lower rates of survival,^23^ or trends toward decreased survival,^24,25^ potentially due to nutritional deficiencies and limited metabolic reserves. Findings regarding obesity are heterogeneous: most studies found no association with mortality,^24,26,27,28^ while others described a protective “obesity paradox,”^29,30,31,32^ possibly due to improved tolerance of catabolism, reduced susceptibility to ventilator-induced lung injury related to obesity-associated altered pulmonary mechanics, and selection bias.^30,33,34^ Other studies have noted increased mortality in adults with obesity,^35,36^ likely attributable to comorbidities, chronic inflammation, or ECMO-related complications.^33,34^ In growing children with obesity, higher metabolic demands and smaller vessel size relative to body weight may impair ECMO flow and worsen outcomes.^21^ Future studies should examine how nutritional status can be incorporated into ECMO candidacy decisions. Given the variability in patient selection across institutions, routine nutritional screening and consideration of BMI thresholds at the time of ECMO evaluation may help clinicians in risk stratification, multidisciplinary decision-making, and individualized patient counseling while helping clarify whether nutritional status should serve as a relative or absolute criterion in candidacy assessments.^37^

Anthropometric and ECMO characteristics followed expected patterns. Patients with underweight were generally younger and more likely to have precannulation hypotension, increased inotropic requirements, prior exposure to CPB, and cardiac indications for ECMO, presumably reflecting the prevalence of postoperative cardiac ECMO in infancy. Conversely, patients with obesity were typically older, more often required ECMO for respiratory indications, and were more likely to receive VV support, possibly due to obesity-related lung dysfunction and increased susceptibility to respiratory infections and ARDS.^38^ Despite these patterns, our subgroup analyses stratified by age and diagnostic group confirmed that the association between BMI *z* score and hospital mortality persisted.

We found that patients with underweight experienced fewer ECMO-related complications, whereas patients with obesity exhibited significantly higher rates of mechanical, pulmonary, and kidney complications. ECMO studies in adults with obesity have reported increased rates of cardiovascular,^29^ kidney,^26,29^ mechanical,^29,39^ hemorrhagic,^27^ infectious,^28^ and limb ischemia^40^ complications. Pediatric data on ECMO complications associated with BMI status are lacking, and to our knowledge, our study is the first to report this association in children, highlighting the need for further research in this population.

Children receiving ECMO face abnormal nutritional demands due to hypermetabolism, protein catabolism, or hypometabolism.^41^ Malnutrition has been associated with increased mortality, complications, and poorer long-term outcomes in this population.^11,41,42,43^ A pilot study^44^ previously used indirect calorimetry to measure resting energy expenditure in children receiving ECMO but identified a high variability in measurements. Previous ECMO literature^42,44^ has not identified a standard method for nutritional assessment, and current technology may not capture the dynamic metabolic alterations occurring in critically ill children receiving ECMO. Optimal nutritional support in pediatric ECMO requires careful balancing.^43^ Early enteral nutrition is preferred to preserve gut integrity, but clinical instability often necessitates parenteral nutrition.^12,13,42,45,46^ Two published surveys of ECMO teams^47,48^ have reported that approximately half of centers routinely use enteral nutrition, and only one-quarter have established ECMO-specific nutritional guidelines. ELSO guidelines recommend baseline nutritional assessments, individualized support, and early enteral nutrition within 48 hours of cannulation or once stable.^49^ Given the complexity of assessing, modifying, and prescribing nutrition during ECMO, recent literature has begun to leverage machine learning approaches, including time series models, to identify optimal nutritional targets in critically ill adults with septic shock.^50^ Similar precision medicine–based methodologies could be applied to optimize nutritional strategies and potentially improve outcomes in patients receiving ECMO. Research in pediatric ECMO nutrition should focus on accurately assessing energy and protein needs while considering underlying conditions, ECMO mode, and concurrent therapies such as KRT. Future investigations should account for comorbid conditions associated with underweight status and distinguish true obesity from fluid accumulation, both of which may adversely affect outcomes. Studies are needed to optimize nutritional composition and identify the safest timing and approach for initiating enteral nutrition while addressing risks of gut ischemia and feeding intolerance. Creating evidence-based standardized protocols can enhance patient outcomes and facilitate evaluation of various nutritional strategies on both short- and long-term outcomes in this high-risk population.

### Limitations

This study has several limitations. As a retrospective registry analysis, it is subject to incomplete data, selection bias, and unmeasured confounders. The multicenter, international nature of the dataset introduces cultural variability in clinical practices, nutrition guidelines, resource availability, and institutional learning curves. We were unable to account for chronic diseases that could contribute to low BMI or fluid overload, which may elevate BMI, both of which may have influenced outcomes in this cohort. The absence of nutritional support data limit our ability to assess the association of specific nutrition strategies with patient outcomes. Last, our limited sample size in patients with obesity and severe obesity underscores the need for further investigation.

## Conclusions

This retrospective cohort study found that both underweight and obesity status were prevalent among children on ECMO and are independently associated with increased hospital mortality. Prospective studies are required to rigorously evaluate the causal relationship between nutritional status and clinical outcomes in this vulnerable population.

## Notes

Supplement 1. **eFigure 1.** Flow Diagram for Patient Selection and Inclusion in the Study **eTable 1.** Sensitivity Analysis Using Different BMI *z* Score Thresholds **eTable 2.** ECMO Discontinuation Reasons per BMI *z* Score Category **eTable 3.** Patient Characteristics by BMI *z* Score Category, Including Obesity (BMI *z* Score 2-3) and Severe Obesity (BMI *z* Score >3) Subgroups **eTable 4.** Patient Characteristics by BMI *z* Score: Comparison of Obesity (BMI *z* Score 2-3) and Severe Obesity (BMI *z* Score >3) Subgroups **eTable 5.** Multivariable Logistic Regression for Mortality to Hospital Discharge, Including Obesity (BMI *z* Score 2-3) and Severe Obesity (BMI *z* Score >3) Subgroups **eTable 6.** Mortality Proportion by Age Group Across BMI *z* Score Categories **eTable 7.** Mortality Proportion by Diagnostic Group Across BMI *z* Score Categories **eFigure 2.** Probability Mortality by Diagnostic Group Across BMI *z* Score Categories Grouped as Underweight, Normal Weight, and Obese

Supplement 2.
Data Sharing Statement
