# Supplementary material for: Body Mass Index and Survival in Children Receiving Extracorporeal Membrane Oxygenation
Source: JAMA Netw Open. 2026 Apr 20;9(4):e266162. doi: 10.1001/jamanetworkopen.2026.6162 (PMC13096981; doi:10.1001/jamanetworkopen.2026.6162)
Supplement: Supplement 1. — eFigure 1. Flow Diagram for Patient Selection and Inclusion in the Study eTable 1. Sensitivity Analysis Using Different BMI z Score Thresholds eTable 2. ECMO Discontinuation Reasons per BMI z Score Category eTable 3. Patient Characteristics by BMI z Score Category, Including Obesity (BMI z Score 2-3) and Severe Obesity (BMI z Score >3) Subgroups eTable 4. Patient Characteristics by BMI z Score: Comparison of Obesity (BMI z Score 2-3) and Severe Obesity (BMI z Score >3) Subgroups eTable 5. Multivariable Logistic Regression for Mortality to Hospital Discharge, Including Obesity (BMI z Score 2-3) and Severe Obesity (BMI z Score >3) Subgroups eTable 6. Mortality Proportion by Age Group Across BMI z Score Categories eTable 7. Mortality Proportion by Diagnostic Group Across BMI z Score Categories eFigure 2. Probability Mortality by Diagnostic Group Across BMI z Score Categories Grouped as Underweight, Normal Weight, and Obese [file jamanetwopen-e266162-s001.pdf]

Supplementary Online Content

Anton-Martin P, Baxelbaum K, Vellore S, O'Neil E, Modem V, Thadani S. Body mass index and survival in children receiving extracorporeal membrane oxygenation. *JAMA Netw Open.* 2026;9(4):e266162. doi:10.1001/jamanetworkopen.2026.6162

eFigure 1. Flow Diagram for Patient Selection and Inclusion in the Study

eTable 1. Sensitivity Analysis Using Different BMI *z* Score Thresholds

eTable 2. ECMO Discontinuation Reasons Per BMI *z* Score Category

eTable 3. Patient Characteristics by BMI *z* Score Category, Including Obesity (BMI *z* Score 2-3) and Severe Obesity (BMI *z* Score >3) Subgroups

eTable 4. Patient Characteristics by BMI *z* Score: Comparison of Obesity (BMI *z* Score 2-3) and Severe Obesity (BMI *z* Score >3) Subgroups

eTable 5. Multivariable Logistic Regression for Mortality to Hospital Discharge, Including Obesity (BMI *z* Score 2-3) and Severe Obesity (BMI *z* Score >3) Subgroups

eTable 6. Mortality Proportion by Age Group Across BMI *z* Score Categories

eTable 7. Mortality Proportion by Diagnostic Group Across BMI *z* Score Categories

eFigure 2. Probability Mortality by Diagnostic Group Across BMI *z* Score Categories Grouped as Underweight, Normal Weight, and Obese

This supplementary material has been provided by the authors to give readers additional information about their work.

© 2026 Anton-Martin P et al. *JAMA Network Open*.

eFigure 1. Flow Diagram for Patient Selection and Inclusion in the Study

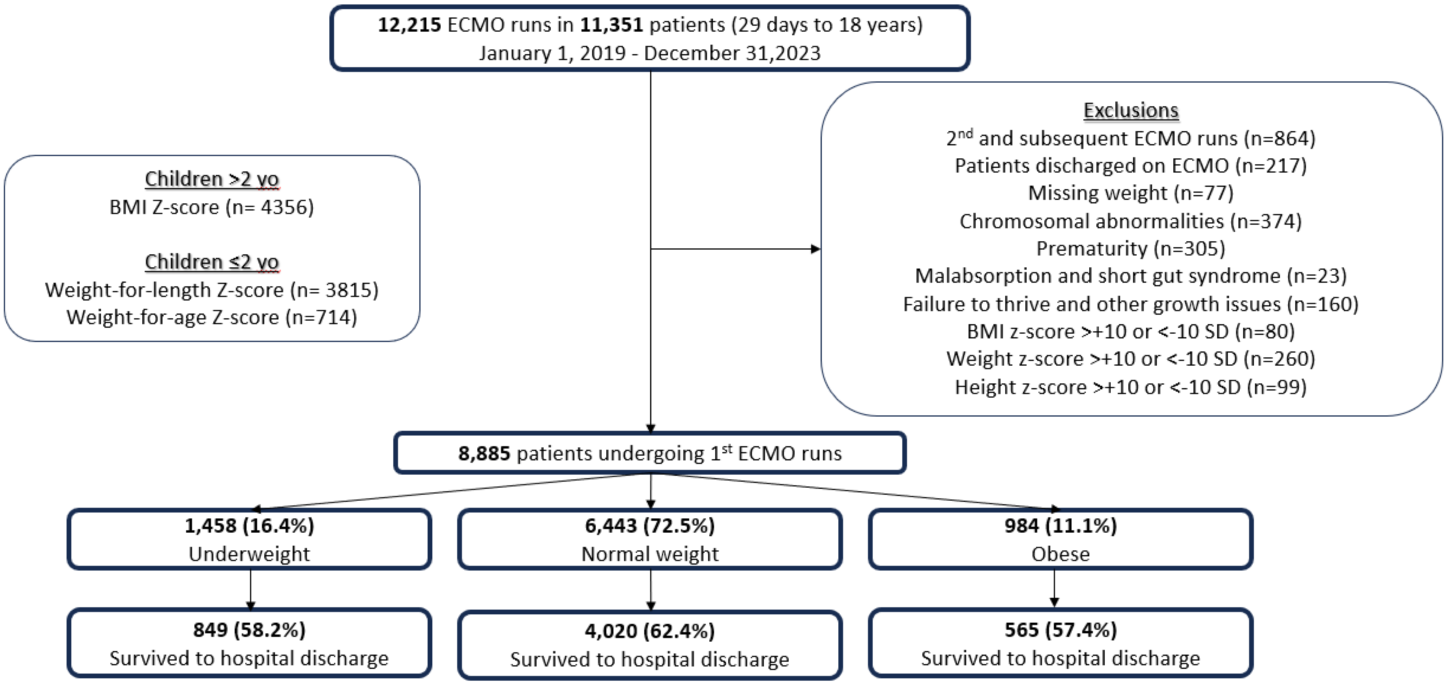

ECMO, extracorporeal membrane oxygenation; BMI, body mass index; SD, standard deviation.

© 2026 Anton-Martin P et al. *JAMA Network Open*.

eTable 1. Sensitivity Analysis Using Different BMI *z* Score Thresholds

Cutoff	±4 SD	±5 SD	±6 SD	±7 SD	±8 SD	±9 SD	±10 SD
Underweight	0.06	**0.03**	**0.01**	**0.004**	**0.003**	**0.005**	**0.005**
Obese	0.15	0.22	0.05	**0.04**	**0.03**	**0.04**	**0.04**
Age	0.28	0.22	0.17	0.16	0.14	0.13	0.14
ARDS	0.48	0.28	0.24	0.19	0.16	0.20	0.20
Non-ARDS resp failure	0.36	0.22	0.17	0.12	0.11	0.15	0.14
Cardiogenic shock	0.30	0.16	0.30	0.29	0.26	0.32	0.32
HF/CMP/Myocarditis/ARR	0.31	0.21	0.19	0.12	0.14	0.20	0.18
Postoperative Cardiac	0.88	0.73	0.59	0.84	0.84	0.75	0.74
Arrest/ECPR	0.13	0.06	0.10	0.07	0.08	0.06	0.06
Sepsis/Infection	0.64	0.89	0.92	0.97	0.98	0.96	0.97
Heme/Onc	0.08	0.08	0.09	0.09	0.09	0.06	0.06
pH	**<0.001**	**<0.001**	**<0.001**	**<0.001**	**<0.001**	**<0.001**	**<0.001**
Hypotension	**<0.001**	**<0.001**	**<0.001**	**<0.001**	**<0.001**	**<0.001**	**<0.001**
CDH	0.05	0.06	0.06	**0.04**	0.06	0.06	0.06
CPB before ECMO	0.05	**0.02**	**0.03**	0.08	0.08	0.08	0.07
HFOV or Prone before ECMO	0.84	0.95	0.94	0.90	0.89	0.77	0.76
Inotropes before ECMO	0.25	0.14	0.07	0.07	0.05	0.05	0.05
Vasopresors before ECMO	0.58	0.61	0.50	0.49	0.38	0.31	0.29
Pulm vasodilators before ECMO	0.35	0.43	0.55	0.44	0.44	0.50	0.54
RRT use before ECMO	**0.04**	**0.02**	**0.002**	**0.003**	**0.005**	**0.002**	**0.002**
VA/VVA	**0.001**	**0.002**	**0.003**	**0.004**	**0.005**	**0.004**	**0.004**
RRT use during ECMO	**<0.001**	**<0.001**	**<0.001**	**<0.001**	**<0.001**	**<0.001**	**<0.001**
ECMO duration	**<0.001**	**<0.001**	**<0.001**	**<0.001**	**<0.001**	**<0.001**	**<0.001**
Presence of any ECMO complication	**<0.001**	**<0.001**	**<0.001**	**<0.001**	**<0.001**	**<0.001**	**<0.001**

ARDS, acute respiratory distress syndrome; ARR, arrhythmia, CDH, congenital diaphragmatic hernia; CMP, cardiomyopathy; CPB, cardiopulmonary bypass; ECMO, extracorporeal membrane oxygenation; ECPR, extracorporeal cardiopulmonary resuscitation; Heme/Onc, hematology/oncology; HF, heart failure; HFOV, high-frequency oscillatory ventilation; Pulm, pulmonary; RRT, renal replacement therapy; SD, standard deviation; VA, veno-arterial; VVA, veno-veno-arterial.

© 2026 Anton-Martin P et al. *JAMA Network Open*.

eTable 2. ECMO Discontinuation Reasons Per BMI *z* Score Category

Characteristic	Overall N = 8,885	Underweight N = 1,458	Normal weight N = 6,443	Obese N = 984	p-value
Died or Poor Prognosis	2,383 (27%)	396 (27%)	1,677 (26%)	310 (32%)	**<0.001**
ECMO Complication	129 (1%)	27 (2%)	89 (1%)	13 (1%)	
Expected Recovery	5,782 (65%)	939 (64%)	4,224 (66%)	619 (63%)	
Transplant	113 (1%)	15 (1%)	89 (1%)	9 (1%)	
— Heart	70 (62%) *	6 (40%) *	56 (63%) *	8 (90%) *	
— Lung	39 (34%) *	8 (53%) *	30 (34%) *	1 (10%) *	
— Heart & Lung	4 (3%) *	1 (7%) *	3 (3%) *	0 (0%) *	
VAD	340 (4%)	46 (3%)	270 (4%)	24 (2%)	
Other/unknown	138 (2%)	35 (2%)	94 (1%)	9 (1%)	

* Proportion of the total transplanted. ECMO, extracorporeal membrane oxygenation; VAD: ventricular assist device

© 2026 Anton-Martin P et al. *JAMA Network Open*.

eTable 3. Patient Characteristics by BMI *z* Score Category, Including Obesity (BMI *z* Score 2-3) and Severe Obesity (BMI *z* Score >3) Subgroups

Characteristic	Overall N = 8,885	Underweight N = 1,458 (17%)	Normal weight N = 6,443 (72%)	Obese N = 573 (6%)	Severely Obese N = 411 (5%)	p-value
**Demographic Data**						
Age (years)	1.9 (0.4, 10.7)	0.6 (0.3, 2.9)	2.4 (0.5, 11.5)	6.5 (0.7, 14.3)	2.8 (0.4, 13.2)	**<0.001**
Age Group						**<0.001**
Infant (29 do-1 yo)	3,590 (40%)	917 (63%)	2,356 (37%)	162 (28%)	155 (38%)	
Pre-school (1-5 yo)	2,175 (24%)	325 (22%)	1,644 (26%)	119 (21%)	87 (21%)	
School-age (6-11 yo)	1,171 (13%)	112 (8%)	908 (14%)	100 (17%)	51 (12%)	
Adolescent (12-18 yo)	1,949 (22%)	104 (7%)	1,535 (24%)	192 (34%)	118 (29%)	
Weight (kg)	11 (6, 36)	6 (4, 12)	12 (7, 39)	28 (9, 90)	17 (7, 112)	**<0.001**
Sex						**0.005**
Female	4,112 (46%)	635 (44%)	3,057 (47%)	244 (43%)	176 (43%)	
Male	4,773 (54%)	823 (56%)	3,386 (53%)	329 (57%)	235 (57%)	
CDH	26 (0.3%)	10 (0.7%)	13 (0.2%)	3 (0.5%)	0 (0%)	**0.01**
Diagnostic group						**<0.001**
ARDS	523 (6%)	62 (4%)	384 (6%)	39 (7%)	38 (9%)	
Non-ARDS resp fail	2,051 (23%)	290 (20%)	1,486 (23%)	149 (26%)	126 (31%)	
Cardiogenic shock	541 (6%)	98 (7%)	386 (6%)	40 (7%)	17 (4%)	
HF/CMP/myoc/ARR	1,180 (13%)	165 (11%)	923 (14%)	56 (10%)	36 (9%)	
Postoperative Cardiac	2,071 (23%)	490 (34%)	1,433 (22%)	82 (14%)	66 (16%)	
Arrest/ECPR	808 (9%)	110 (7%)	605 (9%)	62 (11%)	31 (7%)	
Sepsis/Infection	914 (10%)	103 (7%)	646 (10%)	101 (18%)	64 (16%)	
Heme/Onc	83 (1%)	7 (1%)	68 (1%)	4 (1%)	4 (1%)	
Other/Unknown	714 (8%)	133 (9%)	512 (8%)	40 (7%)	29 (7%)	
**Pre-ECMO Data**						
Ventilation modality						0.2
Conventional	5,669 (64%)	972 (67%)	4,086 (63%)	350 (61%)	261 (64%)	
HFOV	632 (7%)	95 (6%)	455 (7%)	46 (8%)	36 (9%)	
Other	2584 (29%)	391 (27%)	1902 (30%)	177 (31%)	114 (27%)	
pH	7.25 (7.11, 7.35)	7.25 (7.10, 7.34)	7.26 (7.12, 7.35)	7.25 (7.11, 7.35)	7.26 (7.12, 7.36)	0.7
Hypotension	3,213 (44%)	613 (52%)	2,312 (44%)	171 (36%)	117 (36%)	**<0.001**
RRT use pre ECMO	319 (4%)	43 (3%)	236 (4%)	26 (4%)	14 (3%)	0.3
Pulmonary vasodilators	2,229 (25%)	377 (26%)	1,585 (25%)	147 (26%)	120 (29%)	0.2
Inotropes	5,640 (63%)	1,010 (69%)	4,061 (63%)	343 (60%)	226 (55%)	**<0.001**
Vasopressors	3,254 (37%)	544 (37%)	2,377 (37%)	204 (36%)	129 (31%)	0.13

© 2026 Anton-Martin P et al. *JAMA Network Open*.

HFOV or Prone	815 (9%)	130 (9%)	591 (9%)	50 (9%)	44 (11%)	0.7
CPB	1,600 (18%)	345 (24%)	1,148 (18%)	65 (11%)	42 (10%)	**<0.001**
**ECMO Data**						
ECMO type						**<0.001**
Cardiac	4,192 (47%)	768 (53%)	3,057 (47%)	229 (40%)	138 (34%)	
Pulmonary	2,803 (32%)	365 (25%)	2,027 (31%)	225 (39%)	186 (45%)	
ECPR	1,890 (21%)	325 (22%)	1,359 (21%)	119 (21%)	87 (21%)	
ECMO mode						**<0.001**
VA/VVA	6,975 (79%)	1,240 (85%)	5,049 (78%)	410 (72%)	276 (67%)	
VV/Other	1,910 (21%)	218 (15%)	1,394 (22%)	163 (28%)	135 (33%)	
RRT use during ECMO	2,079 (23%)	295 (20%)	1,519 (24%)	155 (27%)	110 (27%)	**0.002**
**ECMO-related Complications**						
Neurological	1,610 (18%)	268 (18%)	1,180 (18%)	91 (16%)	71 (17%)	0.5
Mechanical	2,364 (27%)	312 (21%)	1,771 (27%)	166 (29%)	115 (28%)	**<0.001**
Metabolic	1,092 (12%)	151 (10%)	813 (13%)	73 (13%)	55 (13%)	0.10
Hemorrhagic	1,731 (19%)	268 (18%)	1,303 (20%)	105 (18%)	55 (13%)	**0.003**
Pulmonary	560 (6%)	70 (5%)	420 (6%)	44 (8%)	26 (6%)	**0.04**
Renal	2,317 (26%)	320 (22%)	1,705 (26%)	176 (31%)	116 (28%)	**<0.001**
Cardiovascular	938 (11%)	134 (9%)	689 (11%)	72 (13%)	43 (10%)	0.14
Infectious	71 (0.8%)	14 (1%)	52 (0.8%)	4 (0.7%)	1 (0.2%)	0.6
Limb-related	199 (2%)	17 (1%)	159 (2%)	15 (3%)	8 (2%)	**0.02**
**Outcomes**						
Survival to hospital discharge	5,434 (61%)	849 (58%)	4,020 (62%)	329 (57%)	236 (57%)	**0.002**
ECMO duration (hours)	119 (65, 219)	118 (64, 213)	119 (66, 219)	121 (60, 246)	130 (64, 259)	0.7
MV (days)	13 (7, 22)	14 (8, 25)	12 (7, 21)	13 (7, 23)	14 (8, 24)	**0.001**
LOS (days)	34 (15, 69)	41 (19, 75)	33 (15, 68)	33 (13, 71)	32 (12, 65)	**<0.001**

ARDS, acute respiratory distress syndrome; ARR, arrhythmia, CDH, congenital diaphragmatic hernia; CMP, cardiomyopathy; CPB, cardiopulmonary bypass; do, days old; ECMO, extracorporeal membrane oxygenation; ECPR, extracorporeal cardiopulmonary resuscitation; Heme/Onc, hematology/oncology; HF, heart failure; HFOV, high-frequency oscillatory ventilation; kg, kilograms; LOS, length of stay; MV, mechanical ventilation; myoc, myocarditis; Pulm, pulmonary; resp fail, respiratory failure; RRT, renal replacement therapy; VA, veno-arterial; VVA, veno-veno-arterial; VV, veno-venous; yo, years old.

© 2026 Anton-Martin P et al. *JAMA Network Open*.

eTable 4. Patient Characteristics by BMI *z* Score: Comparison of Obesity (BMI *z* Score 2-3) and Severe Obesity (BMI *z* Score >3) Subgroups

Characteristic	Overall<br>N = 984	Obese<br>N = 573 (58%)	Severely Obese<br>N = 411 (42%)	p-value
**Demographic Data**				
Age (years)	4.4 (0.6, 13.9)	6.5 (0.7, 14.3)	2.8 (0.4, 13.2)	**0.002**
Age Group				**0.006**
Infant (29 do-1 yo)	317 (32%)	162 (28%)	155 (38%)	
Pre-school (1-5 yo)	206 (21%)	119 (21%)	87 (21%)	
School-age (6-11 yo)	151 (15%)	100 (17%)	51 (12%)	
Adolescent (12-18 yo)	310 (32%)	192 (34%)	118 (29%)	
Weight (kg)	24 (8, 95)	28 (9, 90)	17 (7, 112)	0.8
Sex				0.9
Female	420 (43%)	244 (43%)	176 (43%)	
Male	564 (57%)	329 (57%)	235 (57%)	
CDH	3 (0.3%)	3 (0.5%)	0 (0%)	0.3
Diagnostic group				**<0.001**
ARDS	77 (8%)	39 (7%)	38 (9%)	
Non-ARDS resp fail	275 (28%)	149 (26%)	126 (31%)	
Cardiogenic shock	57 (6%)	40 (7%)	17 (4%)	
HF/CMP/myoc/ARR	92 (9%)	56 (10%)	36 (9%)	
Postoperative Cardiac	148 (15%)	82 (14%)	66 (16%)	
Arrest/ECPR	93 (9%)	62 (11%)	31 (7%)	
Sepsis/Infection	165 (17%)	101 (18%)	64 (16%)	
Heme/Onc	8 (1%)	4 (1%)	4 (1%)	
Other/Unknown	69 (7%)	40 (7%)	29 (7%)	
**Pre-ECMO Data**				
Ventilation modality				0.7
Conventional	611 (62%)	350 (61%)	261 (64%)	
HFOV	82 (8%)	46 (8%)	36 (9%)	
Other	291 (29%)	177 (31%)	114 (27%)	
pH	7.25 (7.11, 7.35)	7.25 (7.11, 7.35)	7.26 (7.12, 7.36)	0.7
Hypotension	288 (36%)	171 (36%)	117 (36%)	>0.9
RRT use pre ECMO	40 (4.1%)	26 (4.5%)	14 (3.4%)	0.4
Pulmonary vasodilators	267 (27%)	147 (26%)	120 (29%)	0.2
Inotropes	569 (58%)	343 (60%)	226 (55%)	0.13
Vasopressors	333 (34%)	204 (36%)	129 (31%)	0.2
HFOV or Prone	94 (9.6%)	50 (8.7%)	44 (11%)	0.3
CPB	107 (11%)	65 (11%)	42 (10%)	0.6

© 2026 Anton-Martin P et al. *JAMA Network Open*.

**ECMO Data**				
ECMO type				0.10
Cardiac	367 (37%)	229 (40%)	138 (34%)	
Pulmonary	411 (42%)	225 (39%)	186 (45%)	
ECPR	206 (21%)	119 (21%)	87 (21%)	
ECMO mode				0.2
VA/VVA	686 (70%)	410 (72%)	276 (67%)	
VV/Other	298 (30%)	163 (28%)	135 (33%)	
RRT use during ECMO	265 (27%)	155 (27%)	110 (27%)	>0.9
**ECMO-related Complications**				
Neurological	162 (16%)	91 (16%)	71 (17%)	0.6
Mechanical	281 (29%)	166 (29%)	115 (28%)	0.7
Metabolic	128 (13%)	73 (13%)	55 (13%)	0.8
Hemorrhagic	160 (16%)	105 (18%)	55 (13%)	**0.03**
Pulmonary	70 (7%)	44 (8%)	26 (6%)	0.4
Renal	292 (30%)	176 (31%)	116 (28%)	0.4
Cardiovascular	115 (12%)	72 (13%)	43 (10%)	0.3
Infectious	5 (0.5%)	4 (0.7%)	1 (0.2%)	0.4
Limb-related	23 (2%)	15 (3%)	8 (2%)	0.5
**Outcomes**				
Survival to hospital discharge	565 (57%)	329 (57%)	236 (57%)	>0.9
ECMO duration (hours)	125 (62, 255)	121 (60, 246)	130 (64, 259)	0.6
MV (days)	13 (7, 24)	13 (7, 23)	14 (8, 24)	0.4
LOS (days)	32 (13, 68)	33 (13, 71)	32 (12, 65)	0.3

ARDS, acute respiratory distress syndrome; ARR, arrhythmia, CDH, congenital diaphragmatic hernia; CMP, cardiomyopathy; CPB, cardiopulmonary bypass; do, days old; ECMO, extracorporeal membrane oxygenation; ECPR, extracorporeal cardiopulmonary resuscitation; Heme/Onc, hematology/oncology; HF, heart failure; HFOV, high-frequency oscillatory ventilation; kg, kilograms; LOS, length of stay; MV, mechanical ventilation; myoc, myocarditis, Pulm, pulmonary; resp fail, respiratory failure; RRT, renal replacement therapy; VA, veno-arterial; VVA, veno-veno-arterial; VV, veno-venous; yo, years old.

© 2026 Anton-Martin P et al. *JAMA Network Open*.

eTable 5. Multivariable Logistic Regression for Mortality to Hospital Discharge, Including Obesity (BMI *z* Sscore 2-3) and Severe Obesity (BMI *z* Score >3) Subgroups

Characteristic	OR	Std Error	95% CI	P value
Underweight	1.35	0.11	1.09-1.65	**0.005**
Obese	1.26	0.14	0.95-1.66	0.10
Severely obese	1.24	0.17	0.88-1.71	0.20
Age (in Years)	0.99	0.01	0.97-1.00	0.14
ARDS	0.76	0.21	0.49-1.15	0.20
Non-ARDS resp failure	0.77	0.18	0.55-1.09	0.14
Cardiogenic shock	0.81	0.22	0.52-1.24	0.32
HF/CMP/Myocarditis/ARR	0.77	0.19	0.52-1.13	0.18
Postoperative Cardiac	1.06	0.19	0.73-1.54	0.74
Arrest/ECPR	1.52	0.23	0.98-2.37	0.06
Sepsis/Infection	1.01	0.18	0.70-1.44	0.97
Heme/Onc	2.06	0.39	0.96-4.51	0.06
pH	0.35	0.23	0.22-0.55	**<0.001**
Hypotension	1.39	0.08	1.18-1.63	**<0.001**
CDH	3.64	0.69	1.01-17.08	0.06
CPB	0.80	0.13	0.62-1.02	0.07
HFOV or Prone before ECMO	0.96	0.17	0.76-1.21	0.76
Inotropes before ECMO	1.19	0.09	0.99-1.43	0.05
Vasopresors use before ECMO	1.09	0.08	0.92-1.27	0.29
Pulmonary vasodilators before ECMO	1.05	0.08	0.89-1.24	0.54
RRT before ECMO	1.68	0.17	1.20-2.32	**0.002**
RRT during ECMO	1.50	0.09	1.25-1.78	**<0.001**
ECMO duration	1.00	0.000	1.00-1.00	**<0.001**
Presence of any ECMO complication	2.19	0.09	1.82-2.63	**<0.001**
VA/VVA	1.36	0.11	1.10-1.68	**0.004**

ARDS, acute respiratory distress syndrome; CDH, congenital diaphragmatic hernia; CI, confidence interval; CPB, cardiopulmonary bypass; ECMO, extracorporeal membrane oxygenation; ECPR, extracorporeal cardiopulmonary resuscitation; Heme/Onc, hematology/oncology; HF/CMP/Myocarditis/ARR, heart failure, cardiomyopathy, myocarditis, arrhythmia; HFOV, high-frequency oscillatory ventilation; RRT, renal replacement therapy; VA, veno-arterial; VVA, veno-veno-arterial.

© 2026 Anton-Martin P et al. *JAMA Network Open*.

eTable 6. Mortality Proportion by Age Group Across BMI *z* Score Categories

Age Group	Underweight	Normal weight	Obese
Infant (29 do-1yo)	383/917(0.42)	970/2356(0.41)	146/317(0.46)
Pre-school (1-5yo)	134/325(0.41)	579/1644(0.35)	96/206(0.47)
School-age (6-11yo)	48/112(0.43)	309/908(0.34)	57/151(0.38)
Adolescent (12-18yo)	44/104(0.42)	565/1535(0.37)	120/310(0.39)

eTable 7. Mortality Proportion by Diagnostic Group Across BMI *z* Score Categories

Group	Underweight	Normal weight	Obese
Other/Unknown	55/133(0.41)	188/512(0.37)	33/69(0.48)
ARDS	21/62(0.34)	127/384(0.33)	19/77(0.25)
Non-ARDS-respiratory failure	112/290(0.39)	425/1486(0.29)	99/275(0.36)
Cardiogenic shock	37/98(0.38)	146/386(0.38)	25/57(0.44)
HF/CMP/Myocarditis/ARR	49/165(0.30)	299/923(0.32)	37/92(0.40)
Postoperative Cardiac	206/490(0.42)	578/1433(0.40)	68/148(0.46)
Arrest/ECPR	61/110(0.55)	328/605(0.54)	55/93(0.59)
Sepsis/Infection	63/103(0.61)	292/646(0.45)	77/165(0.47)
Heme/Onc	5/7(0.71)	40/68(0.59)	6/8(0.75)

ARDS, acute respiratory distress syndrome; ECPR, extracorporeal cardiopulmonary resuscitation; Heme/Onc, hematology/oncology; HF/CMP/Myocarditis/ARR, heart failure, cardiomyopathy, myocarditis, arrhythmia.

© 2026 Anton-Martin P et al. *JAMA Network Open*.

eFigure 2. Probability Mortality by Diagnostic Group Across BMI *z* Score Categories Grouped as Underweight, Normal Weight, and Obese

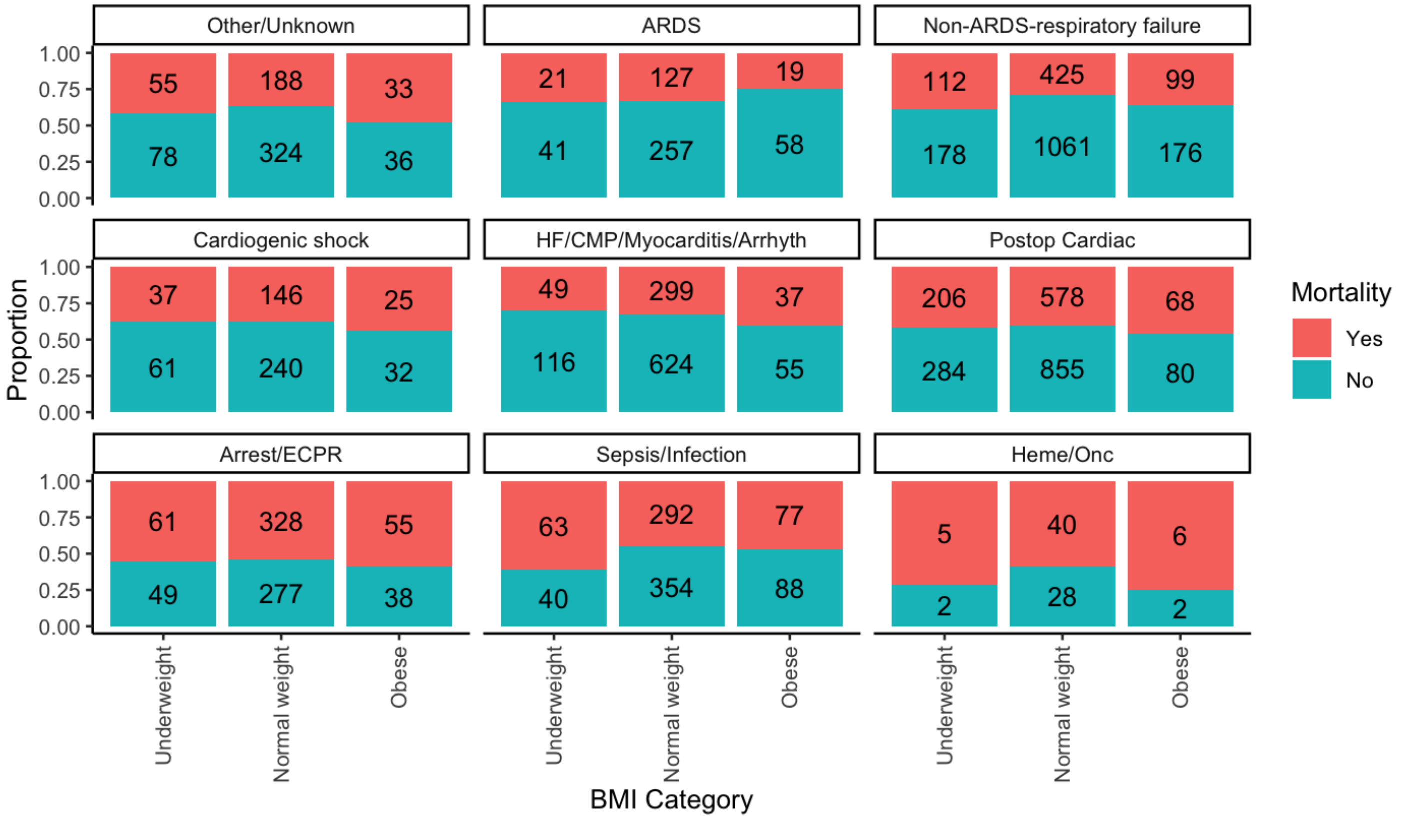

ARDS, acute respiratory distress syndrome; ECPR, extracorporeal cardiopulmonary resuscitation; Heme/Onc, hematology/oncology; HF/CMP/myocarditis/arrhyth, heart failure, cardiomyopathy, myocarditis, arrhythmia; Postop, postoperative.

© 2026 Anton-Martin P et al. *JAMA Network Open*.
