# Supplementary material for: Body Mass Index and Survival in Children Receiving Extracorporeal Membrane Oxygenation
Source: JAMA Netw Open. 2026 Apr 20;9(4):e266162. doi: 10.1001/jamanetworkopen.2026.6162 (PMC13096981; doi:10.1001/jamanetworkopen.2026.6162)
Supplement: Supplement 2. — Data Sharing Statement [file jamanetwopen-e266162-s002.pdf]

Data Sharing Statement

Anton-Martin. Body Mass Index and Survival in Children Receiving Extracorporeal Membrane Oxygenation. *JAMA Netw Open*. Published April 20, 2026.
doi:10.1001/jamanetworkopen.2026.6162

Data
Data available: No

Additional Information
Explanation for why data not available: This data belongs to the ELSO Registry. Requests for data sharing should be submitted directly to ELSO Registry Committee
